# Supplementary material for: Balancing the film strain of organic semiconductors for ultrastable organic transistors with a five-year lifetime
Source: Nat Commun. 2022 Mar 16;13:1480. doi: 10.1038/s41467-022-29221-8 (PMC8927116; doi:10.1038/s41467-022-29221-8)
Supplement: Supplementary file 2 — Supplementary Information [file 41467_2022_29221_MOESM2_ESM.pdf]

Supplementary Information

Balancing the Film Strain of Organic Semiconductors for Ultrastable Organic Transistors with a Five-year Lifetime

Xiaosong Chen,[1#] Zhongwu Wang[1,2#], Jiannan Qi[1#], Yongxu Hu[1], Yinan Huang[1], Shougang Sun[1], Yajing Sun[1], Wenbin Gong[3], Langli Luo[4], Lifeng Zhang[4], Haiyan Du,[5] Xiaoxia Hu,[5] Cheng Han[2], Jie Li[1], Deyang Ji[1], Liqiang Li[1,6,7*], and Wenping Hu[1,7]

[1]Tianjin Key Laboratory of Molecular Optoelectronic Sciences, Department of Chemistry, Institute of Molecular Aggregation Science, Tianjin University, Tianjin 300072, China

[2]SZU-NUS Collaborative Innovation Center for Optoelectronic Science & Technology, International Collaborative Laboratory of 2D Materials for Optoelectronics Science and Technology of Ministry of Education, Institute of Microscale Optoelectronics, Shenzhen University, Shenzhen 518060, China

[3]School of Physics and Energy, Xuzhou University of Technology, Xuzhou, 221018, China

[4]Institute of Molecular Plus, Tianjin University, Tianjin, 300072, China

[5]Analysis and Testing Center of Tianjin University, Tianjin University, Tianjin, 300072, China

[6]Suzhou Institute of Nano-Tech and Nano-Bionics, Chinese Academy of Sciences, Suzhou 215123, China

[7]Joint School of National University of Singapore and Tianjin University, International Campus of Tianjin University, Fuzhou 350207, China

[#]These authors contributed equally to this work

Correspondence and requests for materials should be addressed to L.Q.L.

(E-mail: lilq@tju.edu.cn)

Section 1. Strain analysis by XRD.

For crystalline materials, strain is inevitable and can be from external forces and internal structural defects such as atomic interstitials, dislocations, and vacancies. Therefore, the lattice parameters of crystalline (*d*-spacing) materials are variable due to the existence of strain. For single-crystal materials, the lattice parameters are constant and present almost strain-free due to the periodically arranged atoms/molecules. Taking the strain-free single-crystal material as a reference, the strain value of crystalline materials can be determined as a tensile or compressive state by measuring the lattice parameters. As a typical and high-precision laboratory technique, XRD has a great advantage to characterize the micro- and nano-scale deformation of crystalline materials. The variation in interplanar spacing of crystals is a good parameter to quantitatively describe the lattice deformation and can be easily extracted from the Bragg diffraction peaks of XRD patterns, which is widely used in organic semiconductors, perovskites, and two-dimensional materials.[1-4] Through systematic measurement of XRD patterns, we found that the thin film and thick film suffer from tensile strain and compressive strain compared with the strain-free single-crystal materials, respectively.

To exclude the influence of polymorphism, X-ray diffraction (XRD) analysis was carried out with a peak differentiation and fitting method to separate the thin-film phase (FP) and bulk phase (BP). Because the difference of d-spacing between the thin-film phase and bulk phase of DNTT is very small, the (001) diffraction peak of DNTT film in Fig. 1b is very symmetrical and cannot reflect the polymorphism. Therefore, we further

measured and analysed the diffraction peaks of high orders. At high order peaks, the polymorphism appears from the splitting diffraction peaks. Taking 300-nm DNTT film as an example, the diffraction peaks of (003), (004), and (005) crystal planes are asymmetrical, especially in the (005) crystal plane, which has been completely spitted into two separated peaks (Supplementary Fig. 1). However, for relatively thin films, the intensity of the higher-order peaks after the (003) peak is very weak or even undetectable. Therefore, we finally chose the diffraction peaks of the (003) crystal plane to carry out peak differentiation and fitting analysis of DNTT films with different thicknesses. Consequently, we individually analysed the separated thin-film phase (FP) and bulk phase (BP) and found that the diffraction peaks of both phases shift to a larger diffraction angle with increasing film thickness (Supplementary Fig. 2). The corresponding *d*-spacing of the thin-film phase increases from 1.6294 nm to 1.6345 nm and the bulk phase increases from 1.6178 nm to 1.6243 nm. These results solidly confirm the existence of strain in DNTT films.

Besides, Williamson-Hall (W-H) method was applied to analyse DNTT films with different thicknesses and calculate the strain after excluding the effect of instrument-induced broadening. In the W-H method, it is assumed that the peak broadening in X-ray diffraction data is mainly due to the contribution of grain size and strain after excluding the instrument broadening. Therefore, the broadening of the peaks can be expressed as:

$$\beta_T = \beta_D + \beta_\varepsilon \tag{1}$$

where β_T is the total broadening, β_D is the broadening caused by crystallite size, β_ε is

the broadening caused by the strain. The broadening of the crystallite size can be given by the Scheler equation:

$$D = K\lambda \,/\, \beta_D \cos\theta \tag{2}$$

where D is the grain size, β_D is the full width at half maxima (FWHM) of diffraction peak, and K is the shape factor (K = 0.94). λ is the wavelength of the X-ray source (λ = 0.15405 nm), and θ is the position of the diffraction peak. The X-ray diffraction broadening caused by strain is given by Wilson's formula:

$$\beta_\varepsilon = 4\varepsilon \tan\theta \tag{3}$$

where β_ε the X-ray diffraction broadening caused by the strain, ε is the strain. Thus, combining equations (2) and (3) in equation (1), the W-H equation is usually written as:

$$\beta_T = K\lambda \,/\, D\cos\theta + 4\varepsilon \tan\theta \tag{4}$$

It also can be written as:

$$\beta_T \cos\theta = K\lambda \,/\, D + 4\varepsilon \sin\theta \tag{5}$$

The strain ε can be estimated from the slope of the ($\beta_T$cosθ) vs.(4sinθ) graph (the Williamson-Hall plot). The analysis results (Supplementary Fig. 3) firmly demonstrated the existence of strain in DNTT films.

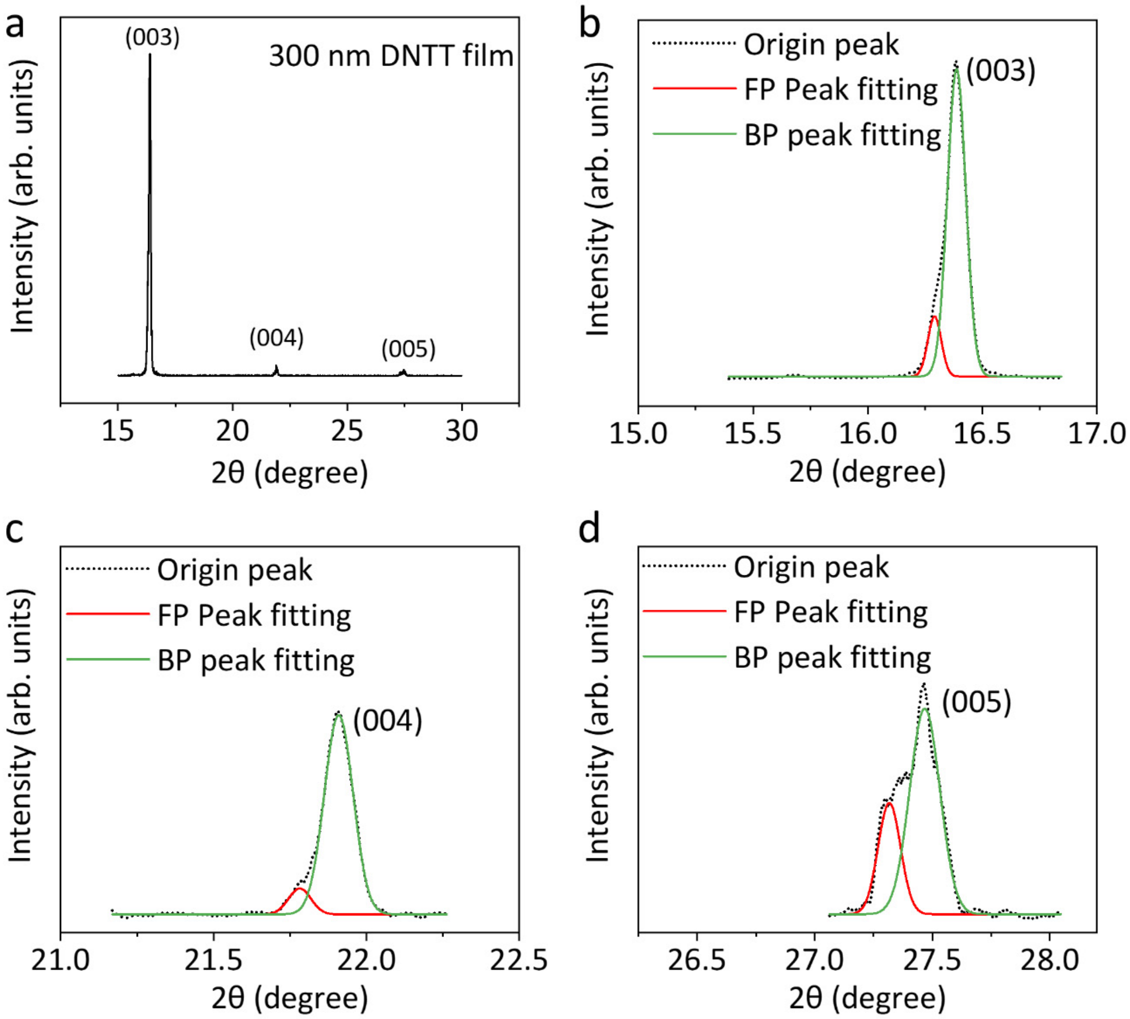

Supplementary Fig. 1 | The XRD patterns of the 300-nm DNTT film and its peak differentiation and fitting results of the (003), (004), and (005) crystal planes.

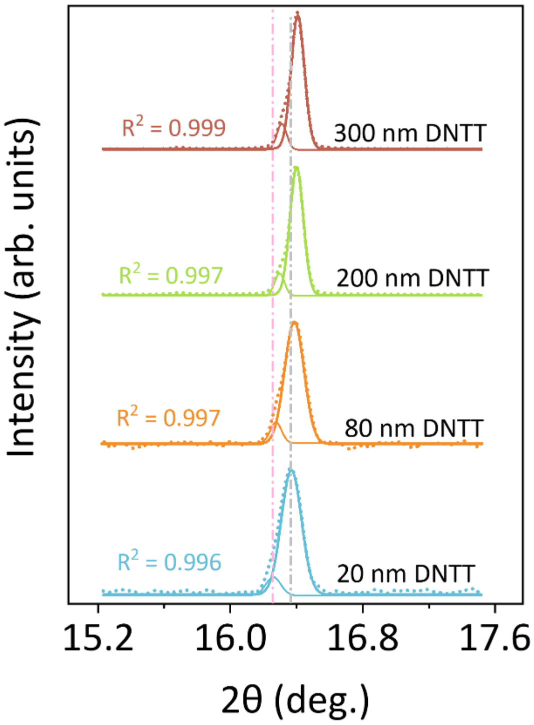

Supplementary Fig. 2 | The peak differentiation and fitting results of the (003) crystal planes of DNTT films with different thicknesses.

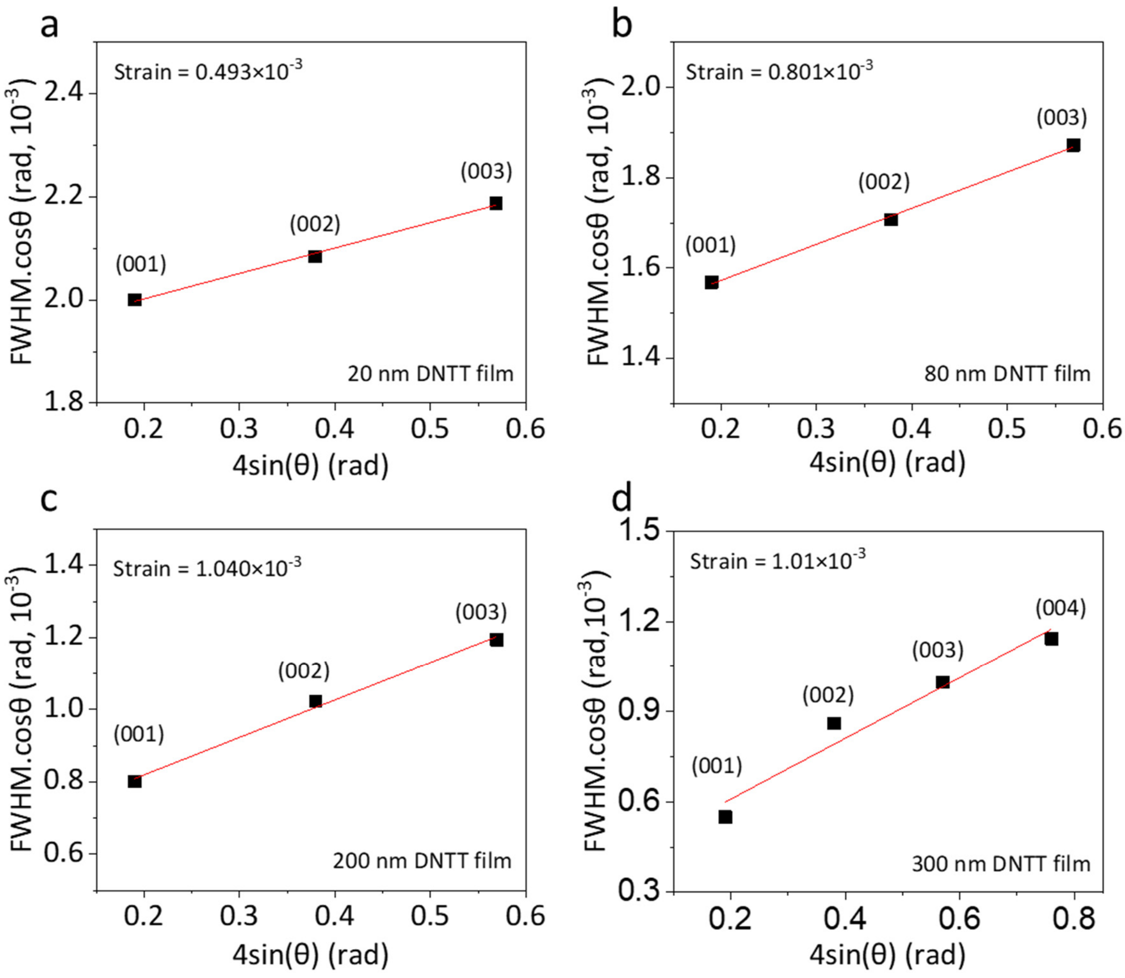

Supplementary Fig. 3 | The strain in DNTT films with different thicknesses by Williamson-Hall (W-H) analysis

Section 2. Aggregate state stability of DNTT films with different thicknesses.

The confocal laser scanning microscopy images with the 200-μm scale for 20-nm and 200-nm thick samples before and after 5 years of storage were shown in Supplementary Fig. 4. It can be seen that the 20-nm DNTT film exhibits severe dewetting phenomenon during storage. The DNTT film (the blue region) aggregated together and the SiO_2 substrate (the dark dots) became visible (enlarged view in Supplementary Fig. 1b). For 200-nm DNTT film (Supplementary Fig. 4c, 4d), the morphology did not show obvious change. The slight color difference of the same samples before and after storage comes from the change of film thickness and the condition of the light source in instruments.

The 300-nm DNTT film has a stable morphology during storage in the microscopic regime (Supplementary Fig. 5), but it forms random microscale cracks and even some macroscale cracks (Fig. 2f). This phenomenon can be understood by the internal stress effect, which demonstrates that the internal stress proportionally depends on the film thickness. The internal stress leads to compressive strain and cracks in DNTT films. Therefore, too thick film (over 300 nm) devices showed a severe performance attenuation during storage due to the appearance of the cracks.

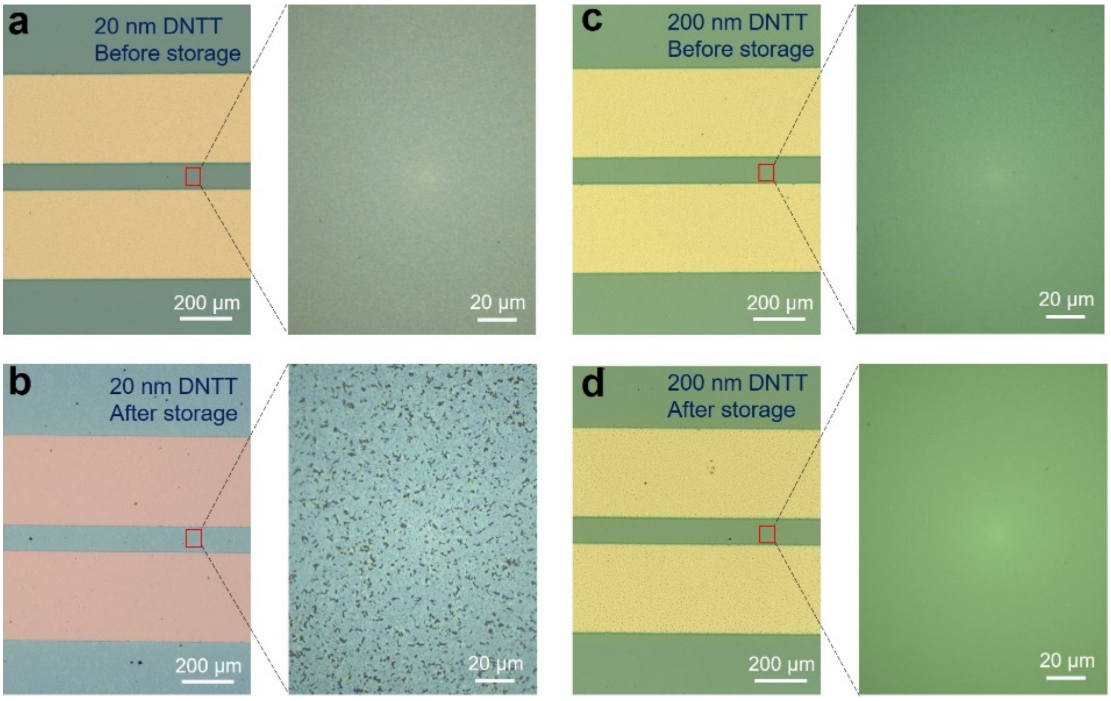

Supplementary Fig. 4 | The confocal laser scanning microscopy images of 20-nm DNTT films (a) before and (b) afterfive years of storage. The confocal laser scanning microscopy images of 200-nm DNTT films (c) before and (d) after five years of storage.

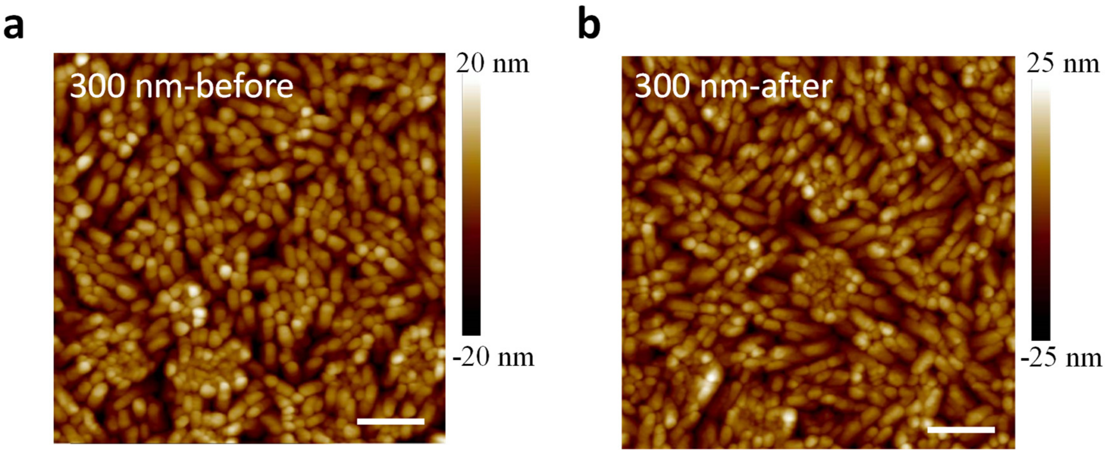

Supplementary Fig. 5 | AFM images of the 300-nm DNTT film (a) before and (b) after five years of storage. The scale bar is 2 μm.

Section 3. Electrical curves of DNTT OFETs with different thicknesses

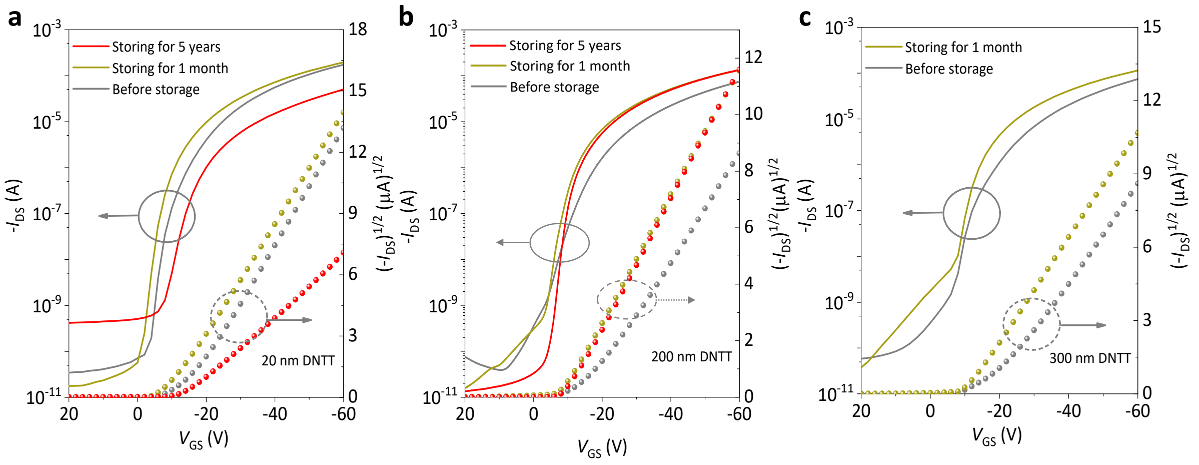

Supplementary Fig. 6 | Transfer curves of OFETs with different thicknesses. (a) 20-nm DNTT. (b) 200-nm DNTT. (c) 300-nm DNTT.

During the storage period, the 20-nm DNTT films suffered from the dewetting problem (Fig. 2b), therefore, their electrical performance underwent severe attenuation (Supplementary Fig. 6a). The 200-nm DNTT films remain a stable aggregate state (Fig. 2d, Supplementary Fig. 4c,d) and have good electrical performance (Supplementary Fig. 6b). However, lots of cracks appeared on the surface of the 300-nm DNTT film (Fig. 2f) and propagate into the conductive channel after about four months (Supplementary Fig. 6c). We are unable to extract the correct parameters from the damaged channel. Therefore, the electrical curves of 300-nm DNTT OFET after four months are not presented here.

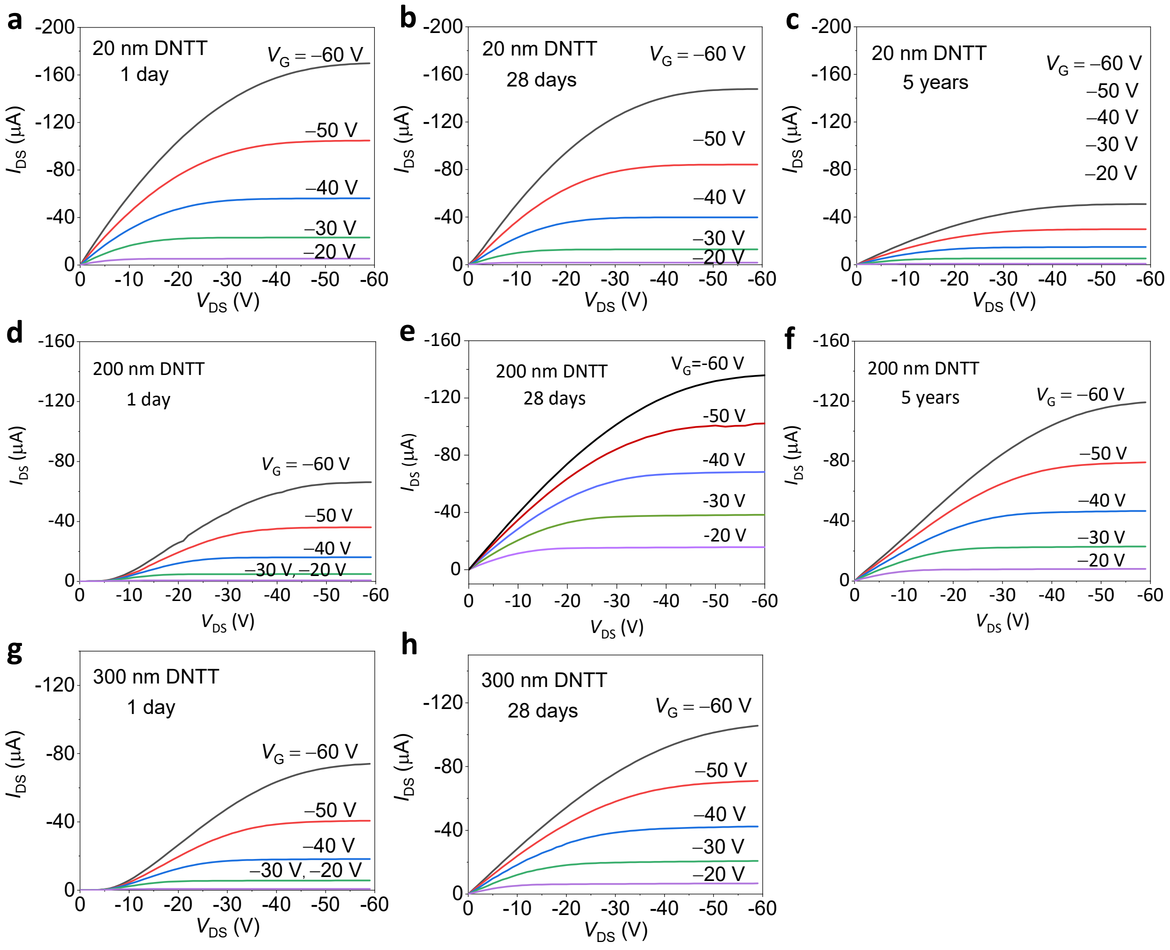

Supplementary Fig. 7 | Output curves of OFETs with different thicknesses. (a-c) 20-nm DNTT. (d-f) 200-nm DNTT. (g, h) 300-nm DNTT. The time in the figures denotes the storage time.

The as-prepared 200-nm DNTT OFET has a high contact resistance and non-linear characteristics (Supplementary Fig. 7d). After being stored for approximately 1 month, the output curves exhibit an ideal ohmic contact due to the gold nanocluster penetration, which is detailly discussed in Section 9. With increasing storage time, the electrical performance remains well even after five years of storage. In contrast, the electrical performance of the thinner and thicker film OFETs significantly attenuates within the storage period.

Section 4. Mobility variation of DNTT OFETs with different thicknesses.

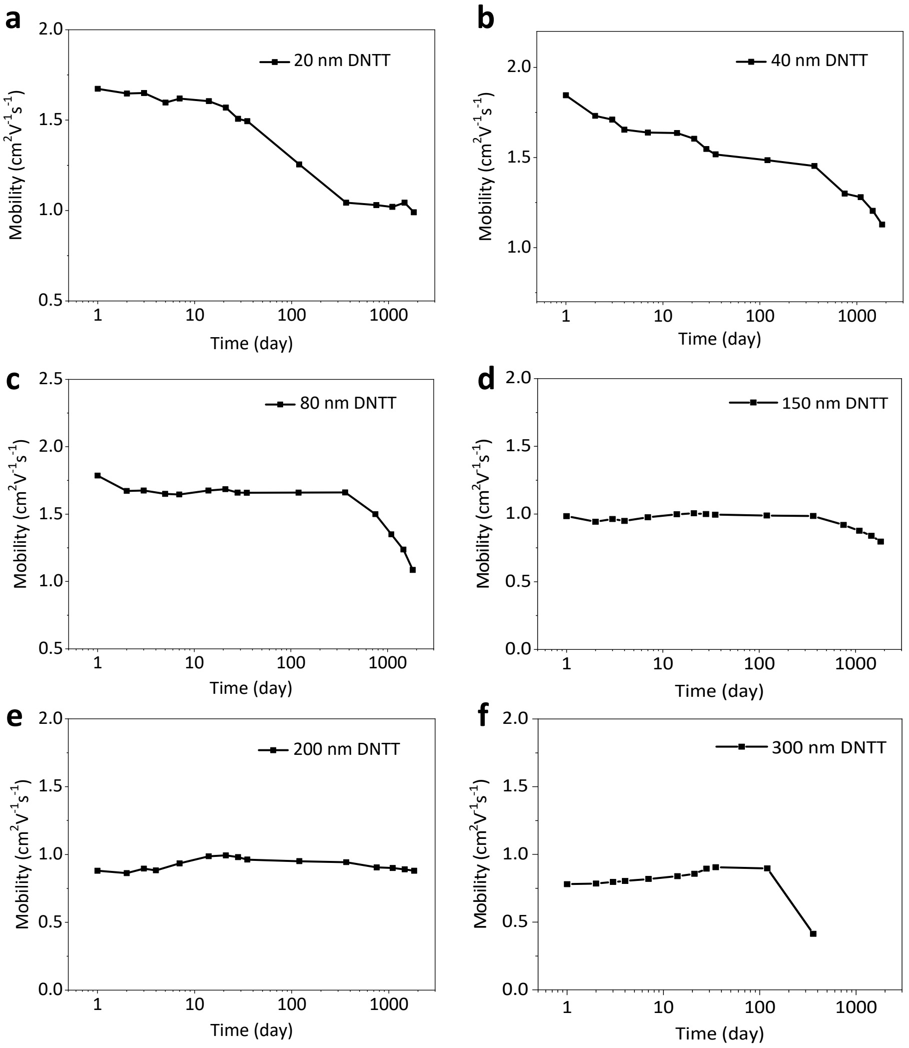

Supplementary Fig. 8 | Mobility variation of DNTT OFETs with different thicknesses. (a) 20-nm DNTT OFET. (b) 40-nm DNTT OFET. (c) 80-nm DNTT OFET. (d) 150-nm DNTT OFET. (e) 200-nm DNTT OFET. (f) 300-nm DNTT OFET.

The mobility variation of DNTT devices with different thicknesses during the five years of storage. The 200-nm DNTT device exhibits the most stable characteristics. Especially, in the first 120 days, mobility stability strictly follows “the thicker the film, the more stable the mobility”. The mobility is influenced by the transport at the semiconductor/dielectric interface and injection at the semiconductor/electrode interface in OFET. In thick film devices, the injection barrier (*i.e.* contact resistance) at

the semiconductor/electrode interface decreases largely at the beginning of storage, which leads to the increase of the calculated mobility during the first month of storage. To show the degradation of the device mobility more objectively, the mobility of the 200-nm DNTT device after five years of storage is compared to that at the first month of storage, corresponding to the highest mobility of the 200-nm DNTT device.

Section 5. Chemical stability of DNTT films.

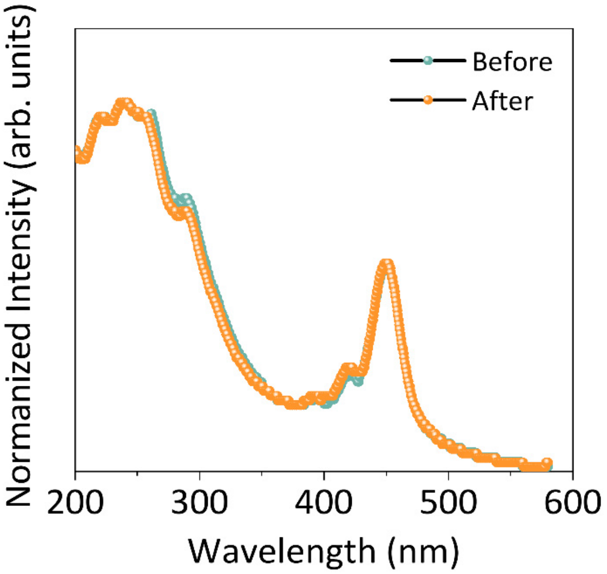

Supplementary Fig. 9 | UV–Vis absorption spectra of DNTT films before and after five years of storage.

Traditionally, the origins of device instability are generally ascribed to chemical component degradation. We characterized the UV–Vis absorption spectra of DNTT film before and after storage (Supplementary Fig. 9). The negligible difference among absorption peaks demonstrates that the chemical structure of DNTT film remains stable even exposed to oxygen, water vapor, and light during storage.

Section 6. The universality of the "strain balance" strategy.

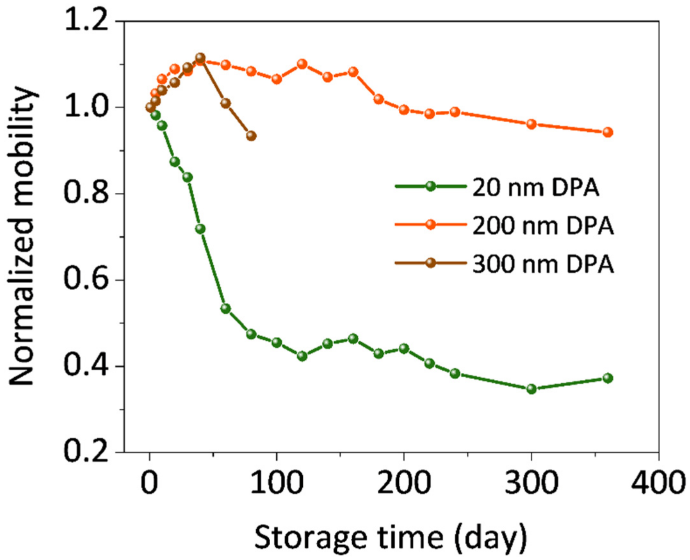

Supplementary Fig. 10 | The variation of the normalized mobility of DPA OFETs with different thicknesses during one year of storage.

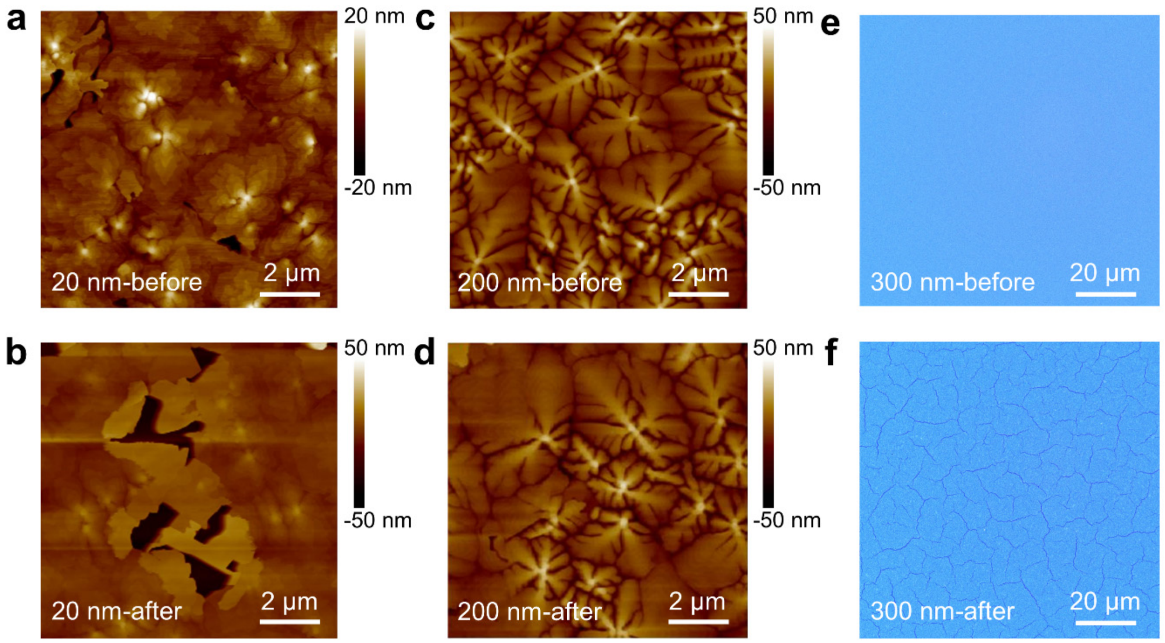

Supplementary Fig. 11 | Morphology evolution of DPA films with different thicknesses. (a-d) Atomic force microscope images of the 20-nm and 200-nm DNTT films before and after five years of storage. The images of 300-nm DPA film (e) before and (f) after five years of storage by confocal laser scanning microscope.

To demonstrate the universality, 2,6-diphenyl anthracene (DPA) films with different thicknesses (20, 200, and 300 nm) were deposited on SiO_2 and stored in the ambient environment for approximately one year. Their mobility (Supplementary Fig. 10) and morphology evolution (Supplementary Fig. 11) present a similar trend as DNTT OFETs, which proves the universality of the “strain balance” strategy for realizing a stable aggregate state and device performance.

Section 7. The self-optimized contact resistance of the 200-nm DNTT OFET.

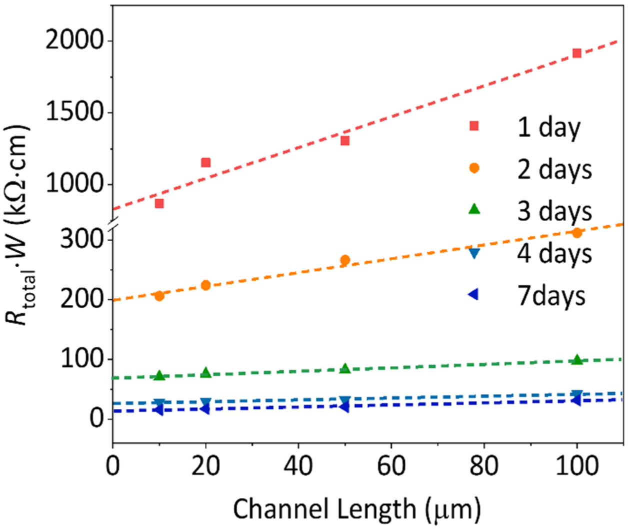

Supplementary Fig. 12 | Plots of the total resistance (R_{total}) versus channel length of the 200-nm DNTT OFET at different times.

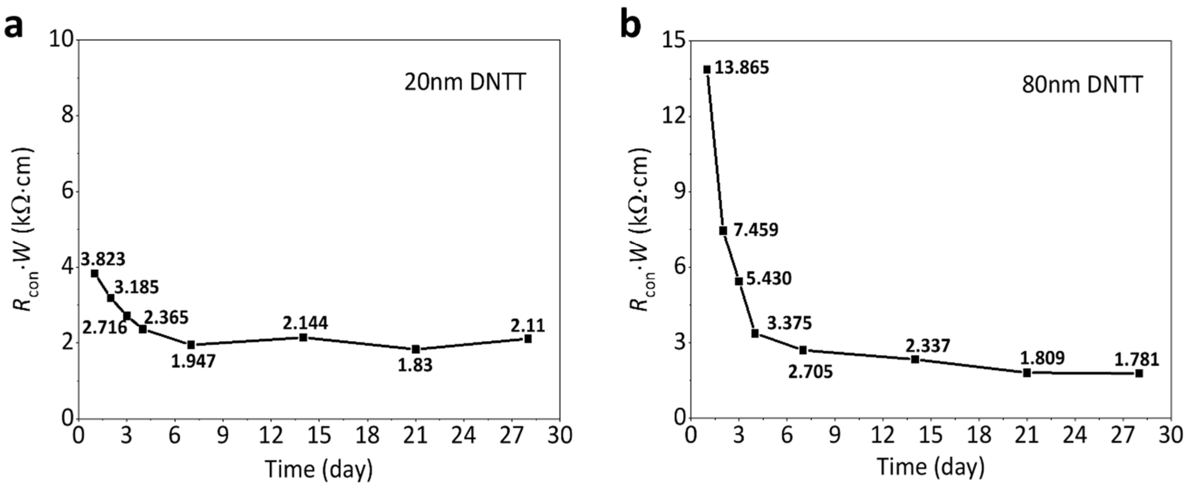

Supplementary Fig. 13 | The normalized contact resistance versus storage time of (a) the 20-nm

DNTT device and (b) the 80-nm DNTT device.

The normalized contact resistance of the 20-nm DNTT OFET keeps stable during storage, while the value of the 80-nm DNTT devices underwent a similar self-optimization process as the 200-nm DNTT device. After storing for approximately one month, the contact resistance of the 200-nm DNTT device (about 800 kΩ·cm) drastically attenuated by two orders of magnitude and remained the same level as thin-film (80 nm) devices.

Section 8. The gate voltage-dependence of mobility of the 200-nm DNTT OFET.

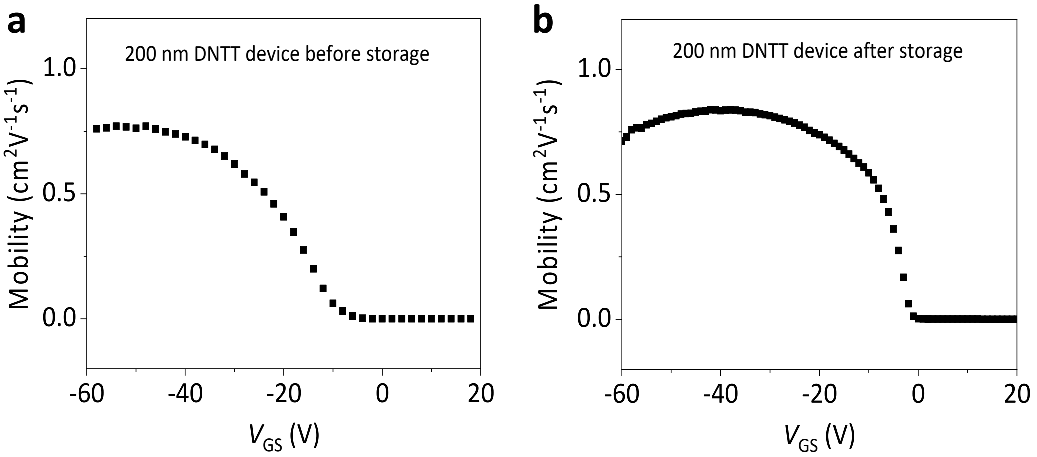

Supplementary Fig. 14 | The gate voltage-dependent mobility of the 200-nm DNTT OFET. (a) Before and (b) after one month of storage.

Although mobility is an intrinsic parameter of organic semiconductors, the extraction of the exact value in OFET is influenced by contact resistance. As shown in Supplementary Fig. 14a, the calculated mobility in OFET with high contact resistance exhibits an obvious dependence on the gate voltage. The higher the contact resistance, the higher the gate voltage-dependence of mobility. After one month of storage (Supplementary Fig. 14b), the mobility shows a slighter gate voltage dependence due to the reduced contact resistance.

Section 9. The origins of the self-optimized contact resistance.

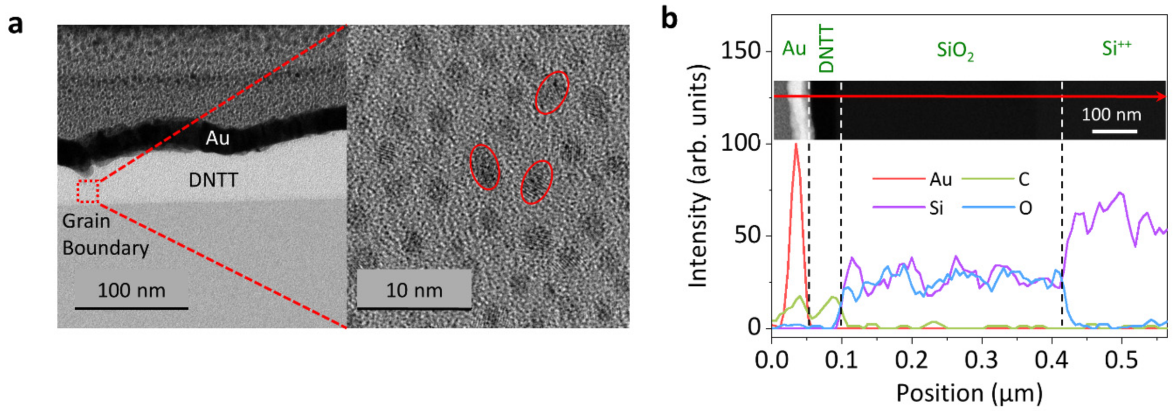

Supplementary Fig. 15 | Structure and component analysis of the charge injection region. (a) High-resolution transmission electron microscope images of the section profile of DNTT OFET. The right panel is the penetrated gold nanoclusters in the DNTT film, and the dark regions marked by red circles are gold nanoclusters. (b) The statistical distribution of elements by the energy dispersive spectrometer (EDS) along the direction from the electrode to the silicon substrate.

As shown in Supplementary Fig. 15a, the cross-sectional view of the high-resolution transmission electron microscope (HRTEM) revealed that some gold nanoclusters penetrate organic semiconductors after electrode deposition. The dark regions marked by red circles are the penetrated gold nanoclusters with diameters within 2~4 nm. Towards the direction from the electrode to the silicon substrate, we detected the statistical distribution of elements by the energy dispersive spectrometer (EDS). Each layer of the device could be clarified by elements distribution. As shown in Supplementary Fig. 15b, the charge injection region (*i.e.*, electrode/semiconductor interface) lies a clear overlap between gold and DNTT, which strongly demonstrates the penetration of gold nanoclusters.

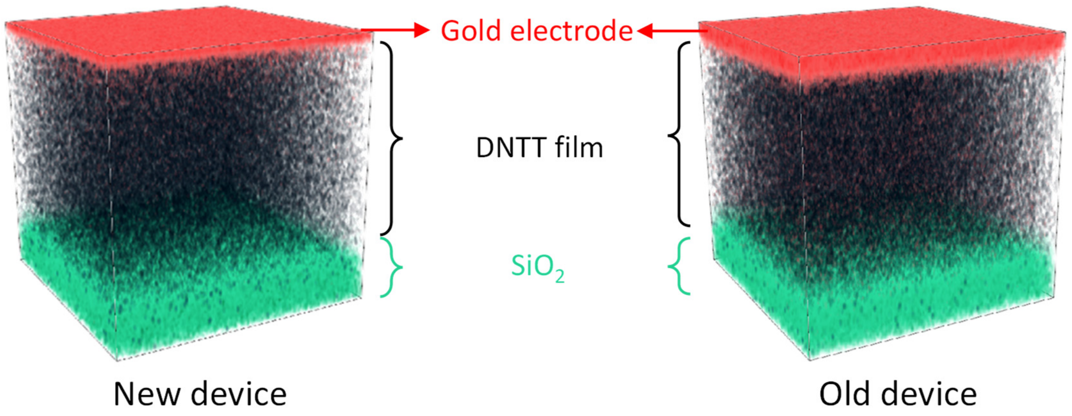

Supplementary Fig. 16 | The element distribution of the 200-nm DNTT device of as-prepared (new device) and after five years of storage (old device).

TOF-SIMS measurements could analyze the element distribution with nanometer precision. To compare the difference of gold electrodes before and after storage, we etched and characterized the device layer by layer from the top electrode to the bottom substrate with a precision of 1 nm. Although some penetrated gold nanoclusters existed in new DNTT film, most gold elements still aggregate at the top region of devices (*i.e.*, source/drain electrode). However, more gold elements exist in a deeper depth of the DNTT film after five years of storage (right panel in Fig. 4d, old device). The penetration depth can reach DNTT/SiO_2 interface (Supplementary Fig. 16). The intensity of each element at a certain etching time can semi-quantitatively denote the element content at different depths, so we plotted the intensity of the elements versus etching time (Fig. 4e). According to the appearing time of each element during the etching process, the component layers of OFET can be distinguished. The orange and blue areas in the inset represent the overlap region between gold and DNTT in the old device and new device, respectively. The Au element of the old device exists at a longer etching time in comparison with the new device, proving a deeper penetration of gold nanoclusters. The schematic diagram of the penetration phenomenon was depicted in Fig. 1a.

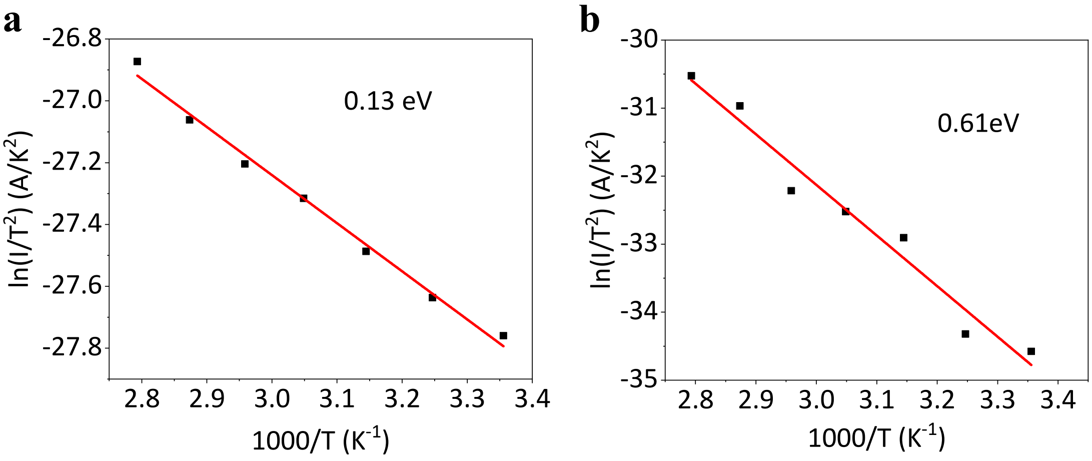

Supplementary Fig. 17 | The effective contact energy barrier. The fitting plot of ln (I/T^2) as a function of 1000/T. (a) Au electrode and DNTT with gold nanoclusters penetration. (b) Au electrode and pure DNTT.

The effective contact energy barrier was evaluated by testing the temperature-dependent characteristic according to the thermionic emission model. The Au electrodes in a pure DNTT/Au reference device are manually attached to DNTT film to avoid gold penetration. As shown in Supplementary Fig. 17, in comparison with the contact energy barrier (0.61 eV) of pure DNTT/Au, the contact energy barrier of DNTT/Au (with gold clusters penetration) decreases to 0.13 eV.

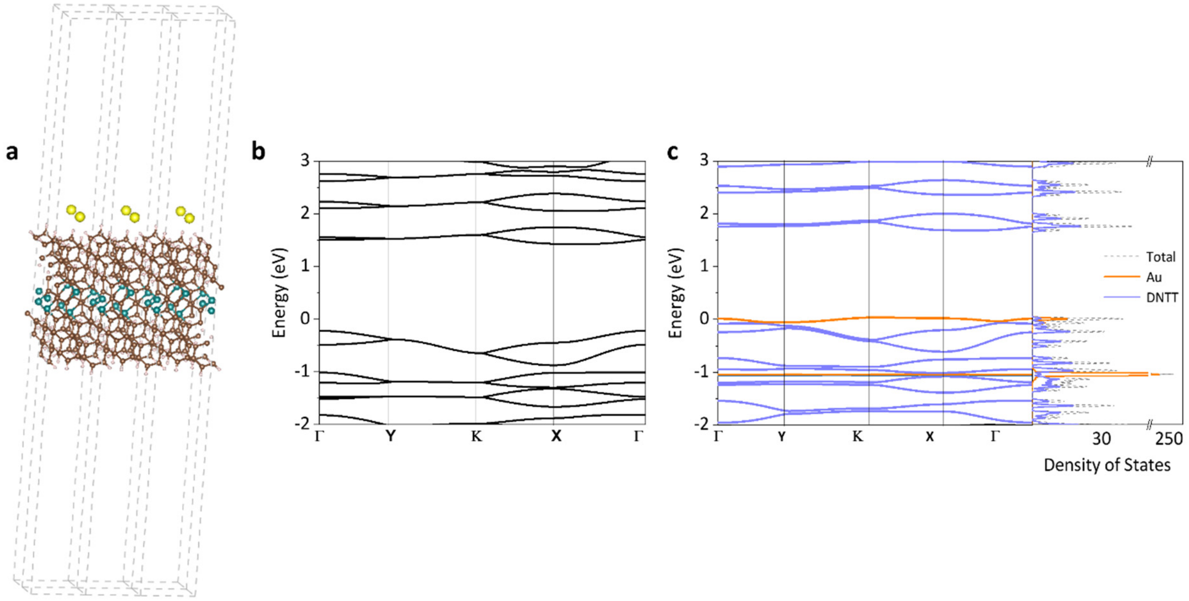

Supplementary Fig. 18 | Theoretical calculation of the contact barrier evolution. (a) The molecular

model of DNTT with Au nanoclusters. Yellow dots are Au atoms. Blue dots are sulfur atoms. Electronic band structure for (b) pure DNTT and (c) DNTT with gold nanoclusters. The right panel in (c) is the density of states.

To further demonstrate the contact barrier evolution, the electronic band structure and density of states of pure DNTT and DNTT with gold nanoclusters were theoretically calculated (Supplementary Fig. 18, calculation details in Experimental Section). The molecular orbitals of DNTT with gold nanoclusters shift upward compared to pure DNTT, which is consistent with the experimental result. The self-optimization process at the electrode/semiconductor interface significantly reduces the contact resistance (Fig. 4c), which is of great importance to overcome the disadvantages of thick film for practical applications.

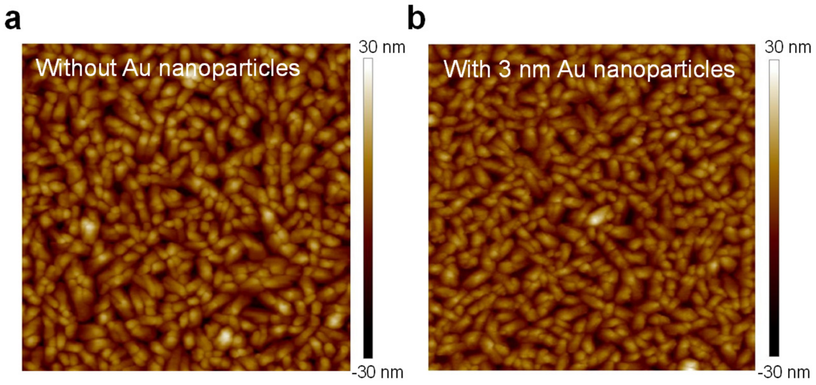

Supplementary Fig. 19｜ AFM images of DNTT film before and after depositing 3-nm gold nanoclusters.

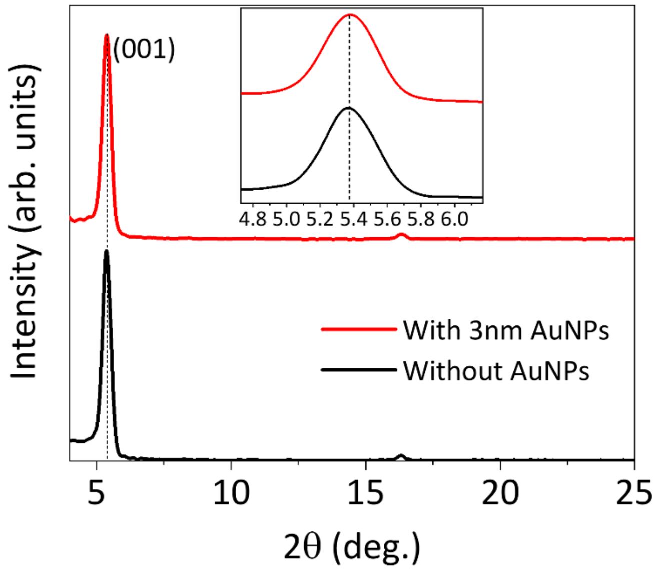

Supplementary Fig. 20 | XRD patterns of DNTT film before and after depositing 3-nm gold nanoclusters.

Section 10. Thickness analysis for different organic semiconductors to produce a stable aggregate state

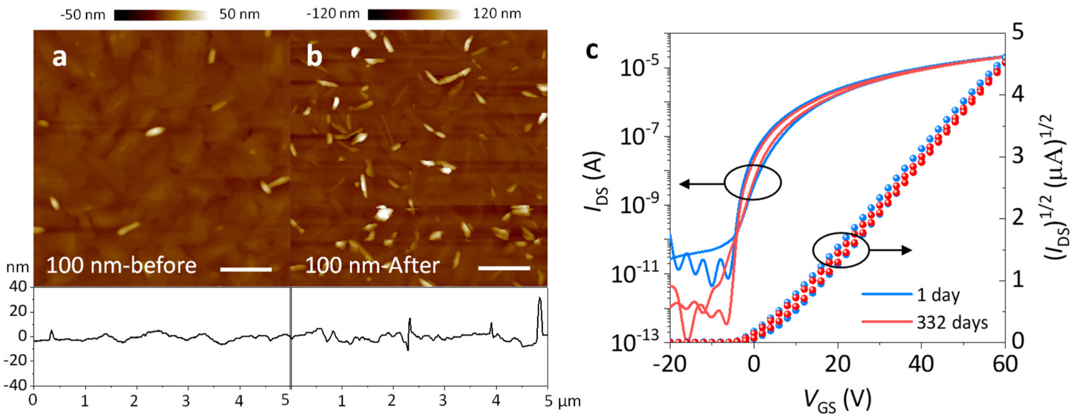

Supplementary Fig. 21 | (a,b) The morphology and (c) transfer curves of PDI-8CN2 OFET before and after 332 days of storage.

Reference

[1] Wang, S. *et al.* Suppressing Interface Strain for Eliminating Double-Slope Behaviors: Towards Ideal Conformable Polymer Field-Effect Transistors. *Adv. Mater.* **33**, 2101633 (2021).

[2] Liu, D. *et al.* Strain analysis and engineering in halide perovskite photovoltaics. *Nat. Mater.* **20**, 1337-1346 (2021).

[3] Xue, D.-J. *et al.* Regulating strain in perovskite thin films through charge-transport layers. *Nat. Commun.* **11**, 1514 (2020).

[4] Wang, M., Li, G., Xu, H., Qian, Y. & Yang, J. Enhanced Lithium Storage Performances of Hierarchical Hollow MoS2 Nanoparticles Assembled from Nanosheets. *ACS Appl. Mater. Interfaces* **5**, 1003-1008 (2013).
